# Synthesis and Luminescent Properties of Dy^3+^-Activated Yellow Phosphors with Anomalous Thermal Quenching for w-LEDs

**DOI:** 10.3390/molecules30234562

**Published:** 2025-11-26

**Authors:** Anlin Zhang, Huapeng Sun, Xiang Li, Bin Deng

**Affiliations:** 1 School of Chemistry and Environmental Science, Xiangnan University, Chenzhou 423043, China; 2 Hunan Provincial Key Laboratory of Xiangnan Rare-Precious Metals Compounds Research and Application, Xiangnan University, Chenzhou 423043, China; 3 Chenjiang Laboratory, School of New Energy, Chenzhou Vocational Technical College, Chenzhou 423000, China

**Keywords:** Dy^3+^, luminescence, w-LEDs

## Abstract

Thermal stability is a crucial factor in evaluating phosphors and determining whether they can be utilized in white light emitting diodes (w-LEDs). In this work, a series of Sr_6_LuAl(BO_3_)_6_: Dy^3+^ (SLAB:Dy^3+^) phosphors was synthesized via high-temperature solid-state reaction. The synthesized SLAB:Dy^3+^ phosphor exhibits narrow-band emission in the range of 450–700 nm under 348 nm UV excitation. The strongest emission peak is located at 577 nm and is primarily due to ^4^F_9/2_-^6^H_13/2_ electron transitions. The optimal doping concentration of Dy^3+^ in the synthesized phosphor was 15 mol%. The integrated emission intensity of the synthesized phosphor at 480 k is 97.84% of that at 300 k, with excellent thermal stability. The activation energy *E*_g_ = 0.62 eV. Meanwhile, the Commission International de l’Eclairage (CIE) coordinates of the prepared w-LEDs were (0.309,0.363) with a correlated color temperature (CCT) of 6497 K. Preliminary experimental findings suggest that SLAB:Dy^3+^ phosphors hold promise for utilization in w-LEDs applications.

## 1. Introduction

In recent decades, with the progress of science and technology, indoor lighting has also undergone radical changes, from the initial incandescent lamps and the development of fluorescent lamps to the most common w-LEDs [1,2,3]. As w-LEDs have the advantages of high luminous efficiency, energy saving, environmental friendliness, and long lifespan, they are rapidly becoming a new generation of lighting source [4,5,6,7,8]. This development has significantly enhanced the quality of life for individuals and communities, becoming a prevalent lighting solution in thousands of households worldwide.

Currently, there are three commercial solutions for achieving w-LEDs. The first method entails the combination of red, green, and blue primary color chips to yield white light [9]. The disadvantages of this method are evident, such as complex drive circuits, high costs, and unstable colors. The second is to activate the yellow YAG:Ce^3+^ phosphor with a blue InGaN chip [10], which has a low color rendering index due to the lack of a red component. Therefore, the most common method is to excite tricolor phosphors—red, green, and blue—with near-ultraviolet chips [11,12]. The w-LEDs prepared with this method offer a range of advantages, including high energy efficiency, low cost, high color rendering index, and long lifespan. Therefore, the development of new phosphors is crucial for realizing high-quality w-LEDs.

At present, most commercial w-LEDs phosphors are made by doping rare earth ions into matrix materials such as borates, phosphates, and silicates. Among the many rare earth ions, Dy^3+^ ions have attracted attention due to their rich energy level structure. The electronic configuration of Dy^3+^ is 4f^9^5s^2^5p^6^. Under ultraviolet (UV) or near-ultraviolet (n-UV) excitation, Dy^3+^-doped phosphors exhibit narrow-band emission in three different colors [13,14]: the ^4^F_9/2_ → ^6^H_15/2_ electronic transition is dominated by blue light (magnetic dipole transition), the ^4^F_9/2_ → ^6^H_13/2_ electronic transition is dominated by yellow light (electric dipole transition), while the ^4^F_9/2_ → ^6^H_11/2_ transition is dominated by red light. The intensity of the magnetic dipole transitions and electric dipole transitions is affected by the symmetry of the environment surrounding the luminescent center. If the intensity of the electric dipole transition is strong, it indicates that the Dy^3+^ ion is in a higher asymmetric ligand position, and vice versa. Currently, Dy^3+^-doped fluorescent materials have been extensively studied and reported, such as Li_3_Gd_3_Te_2_O_12_:Dy^3+^ [15], Na_2_CaSn_2_Ge_3_O_12_:Dy^3+^ [16], Sr_3_MgSi_2_O_8_:Dy^3+^ [17], and Ca_3_(PO_4_)_2_:Dy^3+^ [18].

The matrix material is an important component of phosphors, and its performance determines the quality of the phosphors. Borates have been attracting attention in the field of luminescent materials due to their stable physicochemical properties, high optical thresholds, and low synthesis temperatures. The structural types of borates are diverse, such as BO_3_ (planar triangles), BO_4_ (tetrahedra), and the poly-borate ions B_4_O_7_ and B_6_O_13_. Different types of borates correspond to different crystal field environments and exhibit different luminescent properties in luminescent materials. A large number of borate phosphors have been studied and reported. P. Liang et al. prepared Ba_3_Sc_2_(BO_3_)_4_:Eu^2+^ [19] phosphors by partially substituting Sc ions with Al and Ga ions, which exhibited excellent thermal stability. The w-LEDs devices prepared with this phosphor exhibit a high color rendering index and low color temperature. M. Behera et al. synthesized Eu^3+^-doped Y_2_CaB_10_O_19_ [20] phosphors by a high-temperature solid-state method. The results show that the synthesized phosphor is suitable for the preparation of w-LEDs. Therefore, borates are ideal matrix materials for synthesizing rare earth-doped phosphors.

In this work, a series of SLAB:Dy^3+^ phosphors were successfully synthesized by high-temperature solid-state reactions. The phase purity, surface morphology, elemental distribution, spectral properties, and concentration quenching mechanism were systematically studied. Additionally, w-LEDs devices were fabricated by SLAB:Dy^3+^ phosphors, and their optical performance was tested, laying an experimental foundation for their application in the field of solid-state lighting.

## 2. Results and Discussion

**Figure 1:**
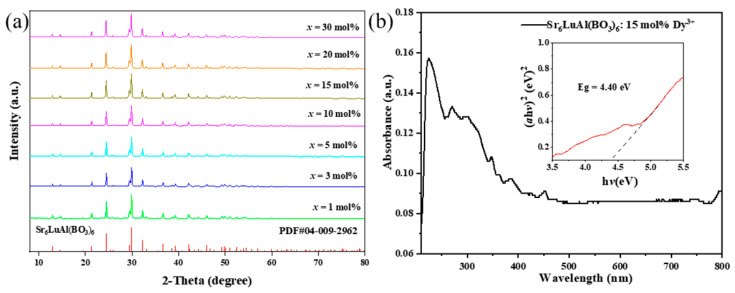
(**a**) XRD patterns of SLAB:*x*Dy^3+^ (*x* = 1–30 mol%). (**b**) UV-vis absorption spectra of SLAB:15 mol% Dy^3+^ (Inset: band gaps of SLAB:15 mol% Dy^3+^).

In previous studies, it has been confirmed that the SLAB host belongs to the hexagonal crystal system with $R\bar{3}$ space group. The lattice parameters of Sr_6_LuAl(BO_3_)_6_ are *a* = *b* = 12.1565 Å, *c* = 9.0853 Å, *V* = 1162.7538 Å^3^ [21]. Figure 1a exhibits the XRD patterns of the prepared SLAB:*x*Dy^3+^ (*x* = 1–30 mol%) phosphors. The XRD curves of phosphors doped with different concentrations of Dy^3+^ ions can be well indexed to the standard card of the SLAB matrix (PDF#04-009-2962) [22]. Compared with standard cards, no additional impurity diffraction peaks were observed in the XRD spectrum, indicating that the incorporation of Dy^3+^ ions did not cause any changes in the crystal structure. Since the atomic radii of Lu^3+^ (r = 0.861 Å) ions and Dy^3+^ (r = 0.912 Å) ions [23,24] are not significantly different, the position of the XRD peak of the phosphors is not affected by the Dy^3+^ ion doping concentration. The percentage difference in the ionic radius (*D*_r_) can be used to determine the substitution site of Dy^3+^ ions in the unit cell which can be calculated from Equation (1) [25]:

(1) $$D_{r}=\frac{\left|Rs\left(CN\right)-Rd\left(CN\right)\right|}{Rs\left(CN\right)}\times 100\%$$

In this equation, *D*_r_ is the percentage difference in ionic radius, whereas CN represents the coordination number. *R*_s_ denotes the radius of the cation in the matrix and *R*_d_ is the radius of the dopant ion. When CN = 6, the radius of the dopant ion *R*_Dy_ = 0.912 Å and the radii of the cations in the matrix are *R*_Lu_ = 0.861 Å, *R*_Al_ = 0.535 Å, and *R*_Sr_ = 1.18 Å, respectively. The *D*_r_ is counted to be 5.92%, 70.47%, and 22.71%, when substituted ions are Lu^3+^, Al^3+^, and Sr^2+^, respectively. Compared with Sr^2+^ and Al^3+^ ions, Lu^3+^ ions have lower *D*_r_ values. Therefore, Dy^3+^ ions preferentially occupy Lu^3+^ ion sites. To study the light absorption capacity of Dy^3+^ phosphors, Figure 1b shows the UV-vis absorption spectrum of SLAB:15 mol% Dy^3+^. The results in the figure show that the SLAB:15 mol% Dy^3+^ sample absorbs photon energy in the range of 220–460 nm. According to the Kubelka–Munk method [26,27], the calculated bandgap energy *E*_g_ = 4.40 eV, as shown in the inset of Figure 1b. The bandgap of the SLAB:Dy^3+^ phosphor is higher than that of the reported Cs_2_NaGdCl_6_:In^3+^ (*E*_g_ = 4.15 eV), Ba_9_Lu_2_Si_6_O_24_:Sm^3+^ (*E*_g_ = 3.95 eV), and La_1.96_LiNbO_6_:0.04Dy^3+^ (*E*_g_ = 4.20 eV) phosphors [28,29,30].

**Figure 2:**
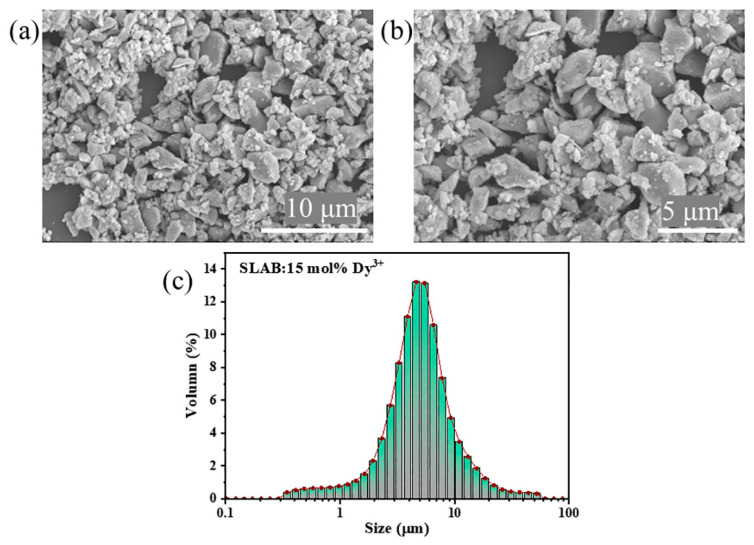
(**a**,**b**) The SEM micrographs of SLAB:15 mol% Dy^3+^ at 4000× and 6000× magnification. (**c**) Particle size distribution of SLAB:15 mol% Dy^3+^ phosphor.

The surface morphology of the SLAB:15 mol% Dy^3+^ phosphor was studied by scanning electron microscopy (SEM). Figure 2a,b shows microscopic images magnified 4000× and 6000×, respectively. SEM results confirm that the synthesized SLAB:15 mol% Dy^3+^ phosphor is irregular in shape and vary in size, which is consistent with the basic characteristics of high-temperature solid-phase reactions. The particle size distribution of the SLAB:15 mol% Dy^3+^ phosphor (Figure 2c) is approximately 5.5 μm, indicating that this phosphor is highly suitable for the fabrication of w-LEDs. However, due to the irregular surface morphology of the SLAB:15 mol% Dy^3+^ sample, further grinding and screening are required during w-LEDs applications to ensure optimal performance.

**Figure 3:**
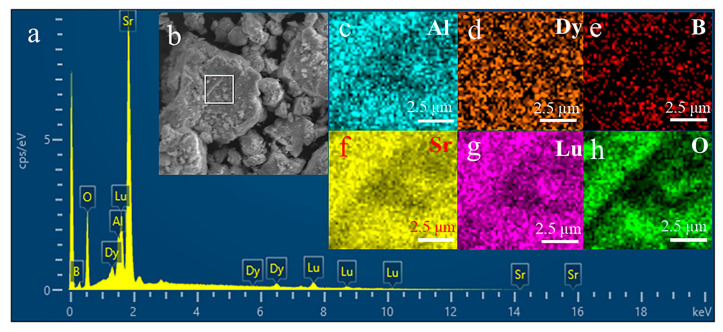
(**a**) The EDS of SLAB:15 mol% Dy^3+^ sample. (**b**–**h**) The original SEM image of the scanned area and the element distribution map maps.

The elemental distribution results (Figure 3a) indicate that the synthesized SLAB:15 mol% Dy^3+^ phosphor contains six elements: B, O, Al, Lu, Sr, and Dy. The weights and atomic percentages of all elements are presented in Table 1. The atomic ratio of O, B, Al, Lu, Sr, and Dy is 52.30:25.45:3.18:2.80:15.85:0.42, which is close to the stoichiometric ratio of the sample. The section within the square frame in Figure 3b represents the original SEM scanning area. Figure 3c–h clearly shows that all elements are evenly distributed in the phosphor. This further confirms that the SLAB:Dy^3+^ phosphor has been successfully synthesized.

**Figure 4:**
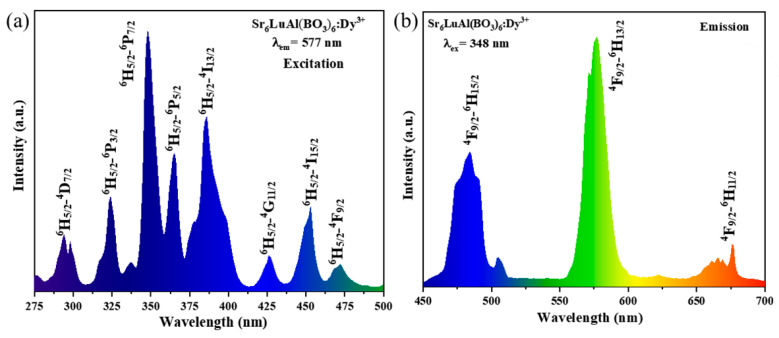
(**a**) Excitation and (**b**) emission spectra of SLAB:15 mol% Dy^3+^ phosphor.

Figure 4a shows the excitation spectrum of the SLAB:15 mol% Dy^3+^ phosphor. Under monitoring at a wavelength of 577 nm, the SLAB:15 mol% Dy^3+^ phosphor exhibits excitation peaks mainly at 294, 324, 348, 365, 386, 426, 453, and 471 nm, corresponding to ^6^H_5/2_ → ^4^D_7/2_, ^6^H_5/2_ → ^6^P_3/2_, ^6^H_5/2_ → ^6^P_7/2_, ^6^H_5/2_ → ^5^P_7/2_, ^6^H_5/2_ → ^4^I_13/2_, ^6^H_5/2_ → ^4^G_11/2_, ^6^H_5/2_ → ^4^I_15/2_, ^6^H_5/2_ → ^4^F_9/2_, respectively [31]. These excitation peaks result from *f*-*f* electronic transitions of Dy^3+^ ions. The strongest excitation peak at 348 nm indicates that SLAB:Dy^3+^ phosphors can be combined with n-UV chips to fabricate w-LEDs. Figure 4b shows the emission spectrum of the SLAB:15 mol% Dy^3+^ phosphor. Under excitation by 348 nm, the SLAB:15 mol% Dy^3+^ sample exhibits three characteristic emission peaks at 484, 577, and 676 nm, corresponding to energy level transitions ^4^F_9/2_ → ^6^H_15/2_, ^4^F_9/2_ → ^6^H_13/2_, and ^4^F_9/2_ → ^6^H_11/2_, respectively [32]. It is well known that magnetic dipole transitions (MD) are almost unaffected by the environment surrounding the matrix activation ions. In contrast, electric dipole (ED) transitions are highly sensitive and only emit at low symmetry sites. In this work, ^4^F_9/2_ → ^6^H_15/2_ and ^4^F_9/2_ → ^6^H_13/2_ correspond to magnetic dipole transitions and electric dipole transitions, respectively. It is clear that the electric dipole transition intensity is much higher than that of the magnetic dipole transition. Therefore, Dy^3+^ ions are located at low symmetry sites in the SLAB matrix [33,34].

**Figure 5:**
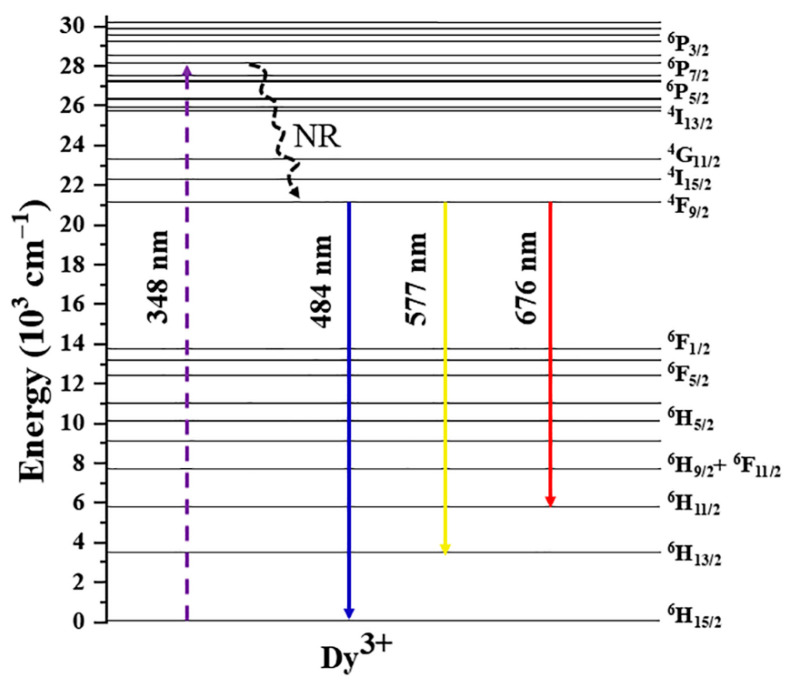
Simplified energy levels scheme of SLAB:15 mol% Dy^3+^.

To better investigate the luminescence mechanism of Dy^3+^ ions, Figure 5 shows the electronic transition energy level diagram. When the synthesized SLAB:15 mol% Dy^3+^ phosphor was excited at 348 nm, the Dy^3+^ ions absorb the energy of the photons, and the electrons in the ground state energy level (^6^H_5/2_) transition to the excited state energy level (^6^P_7/2_). Excited state electrons release part of their energy through non-radiative transitions and return to the ^4^F_9/2_ energy level. Finally, electrons in the ^4^F_9/2_ energy level return to the ground state ^6^H_15/2_, ^6^H_13/2_, and ^6^H_11/2_ energy levels through radiative transitions, corresponding to blue (484 nm), yellow (577 nm), and red (676 nm) light, respectively.

**Figure 6:**
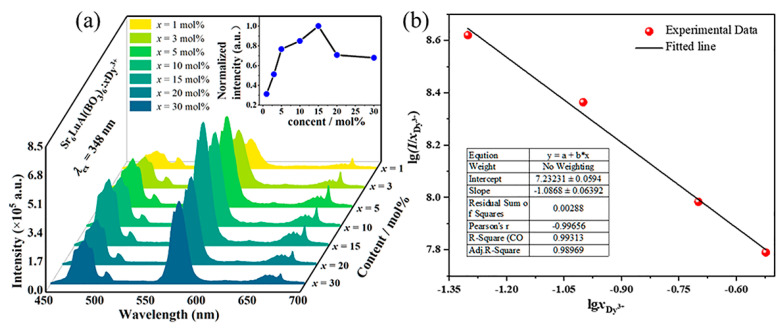
(**a**) Effect of Dy^3+^ ion doping concentration on luminescence intensity. (**b**) The ${\mathrm{lg}}_{x_{Dy^{3+}}}$ − $\mathrm{lg}\left(I/x_{Dy^{3+}}\right)$ curve of SLAB:*x*Dy^3+^.

Dy^3+^ ions serve as the luminescent centers of phosphors, and their doping concentration affects the luminescence intensity of the phosphors. Therefore, determining the optimal doping concentration is beneficial for the application of SLAB:Dy^3+^ phosphors in w-LEDs. Figure 6a shows the effect of Dy^3+^ ion doping concentration on luminescence intensity. The shape and position of the emission peaks of SLAB:Dy^3+^ phosphors are not affected by the doping concentration of Dy^3+^ ions. However, the luminescence intensity is gradually enhanced with the increase in Dy^3+^ ion doping concentration. When the doping concentration reaches 15 mol%, the luminescence intensity reaches the maximum. Beyond this doping concentration, the luminescence intensity gradually decreases due to the concentration quenching.

Concentration quenching involves two mechanisms: electrical multipole interactions and exchange interactions. The critical distance *R*_c_ proposed by Blasse and Grabmaier can distinguish between these two mechanisms. The calculation expression for *R*_c_ is as follows in Equation (2) [35]:

(2) $$R_{c}\approx 2{\left(\frac{3V}{4\pi x_{c}Z}\right)}^{\frac{1}{3}}$$

Here, *V* is the volume of the unit cell, *x*_c_ is the optimal doping concentration of Dy^3+^ ions, and *Z* is the number of Dy^3+^ cations replacing the original cations in each cell. In this work, *V* = 1162.7538 Å^3^, *x*_c_ = 15 mol%, and *Z* = 4. The critical distance *R*_c_ = 7.19 Å is obtained according to Equation (2). If the critical distance *R*_c_ < 5 Å, energy is transferred between different luminescent centers in SLAB:Dy^3+^ phosphors through exchange interactions. Conversely, if the critical distance *R*_c_ > 5 Å, the energy transfer between the luminescent centers is a multipolar interaction. Since the critical distance *R*_c_ of synthetic phosphors is greater than 5 Å, energy transfer multipolar interactions between luminescent centers are the main cause of concentration quenching.

Equation (3) [36,37] is a reliable tool for further studying the concentration quenching mechanism of SLAB:*x*Dy^3+^ phosphors and clarifying the process of energy transfer in the luminescent center.

(3) $$\frac{I}{x}=K{\left(1+\beta {\left(x\right)}^{\frac{Q}{3}}\right)}^{-1}$$

where *x* is the doping concentration of Dy^3+^ ions; *I* is the luminescence intensity; *β* and *K* are constants related to SLAB matrix. Different values of *Q* correspond to different multilevel interactions, namely (*Q* = 3) nearest-neighbor ion interaction, (*Q* = 6) dipole–dipole interaction, (*Q* = 8) dipole–quadrupole interaction, and (*Q* = 10) quadrupole–quadrupole interaction [36]. The variation in ${\mathrm{lg}}_{x_{Dy^{3+}}}$ and $\mathrm{lg}\left(I/x_{Dy^{3+}}\right)$ at 348 nm excitation is presented in Figure 6b. Based on the slope of the fitted curve, the value of *Q* is obtained to be approximately 3.26, which is closest to 3. This indicates that the energy transfer models of the luminescent center in SLAB:Dy^3+^ phosphors are nearest-neighbor ion interactions.

**Figure 7:**
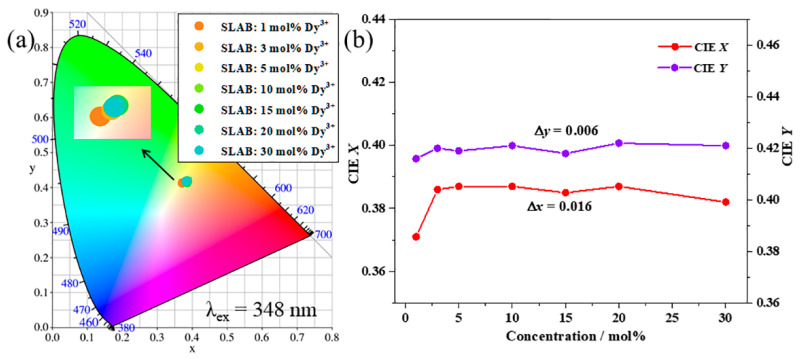
(**a**) Chromaticity coordinates of SLAB:*x*Dy^3+^ sample. (**b**) CIE variation in SLAB:*x*Dy^3+^ samples.

Figure 7a illustrates the CIE chromaticity coordinates of SLAB:*x*Dy^3+^ samples. Obviously, the CIE chromaticity coordinates of phosphors with different Dy^3+^ doping concentrations are all concentrated in the yellow range, as clearly demonstrated by the enlarged illustration. The CIE chromaticity coordinates of SLAB:*x*Dy^3+^ (*x* = 1–30 mol%) are (0.371, 0.416), (0.386, 0.420), (0.387, 0.419), (0.387, 0.421), (0.385, 0.418), (0.387, 0.422), and (0.382, 0.421), respectively. Figure 7b exhibits the CIE variations in SLAB:*x*Dy^3+^ phosphors. The CIE chromaticity coordinates of the SLAB:*x*Dy^3+^ phosphor change slightly with changes in the concentration of the Dy^3+^ ion doping. Such a small change in CIE chromaticity coordinates indicates that the SLAB:*x*Dy^3+^ phosphors possess excellent color stability [38,39].

The CCT of SLAB:*x*Dy^3+^ phosphors (*x* = 1–30 mol%) can be obtained by Equation (4) [40]:

(4) $$CCT=\;-449n^{3}+3625n^{2}-6823.3n+5520.33$$

where *n* equals $\left(x-x_{e}\right)/\left(y-y_{e}\right)$ $\left(x-x_{e}\right)/\left(y-y_{e}\right)$ $\left(x_{e}=0.332,y_{e}=0.186\right)$. By means of the formula, the calculated CCT is 4478, 4136, 4107, 4101, 4157, 4126, and 4227 K, respectively. The CCT of SLAB:*x*Dy^3+^ phosphor varies with the concentration of Dy^3+^ ion doping. Based on the above research findings, SLAB:*x*Dy^3+^ phosphors demonstrate potential application value in w-LEDs.

**Figure 8:**
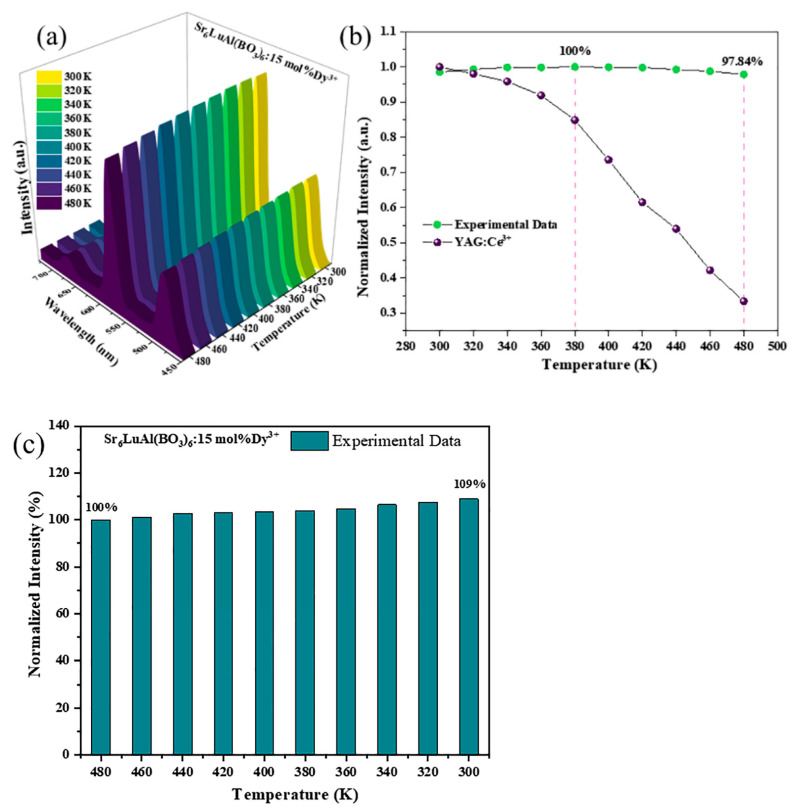
(**a**) Temperature-dependent photoluminescence spectrum of SLAB:*x*Dy^3+^ phosphor (*λ*_ex_ = 348 nm). (**b**) Comparison chart of thermal stability with YAG phosphor. (**c**) The normalized cooling emission intensity of SLAB:15 mol% Dy^3+^ from 480 K to 300 K.

Excellent thermal stability is one of the key indicators for the application of phosphors in w-LEDs. Figure 8a shows the curve of the luminescence intensity of SLAB:15 mol% Dy^3+^ phosphor as a function of temperature. During the temperature rise from 300 K to 480 K, the shape and position of the emission peaks of SLAB:15 mol% Dy^3+^ phosphor remain unchanged, indicating that the phosphor has excellent thermal stability. Figure 8b shows a comparison of the thermal stability of SLAB:15 mol% Dy^3+^ phosphor and YAG phosphor. The comparison diagram clearly shows that at 380 K, the emission intensity of the SLAB:15 mol% Dy^3+^ phosphor is slightly higher than the intensity at 300 K, while the YAG phosphor only retains 84.83%. More importantly, when the temperature reaches 480 K, the luminous intensity of the SLAB:15 mol% Dy^3+^ phosphor remains as high as 97.84%, while that of the YAG phosphor drops to only 33.41%. The comparison results show that the thermal stability of the SLAB:15 mol% Dy^3+^ phosphor is far superior to that of the YAG phosphor. The operating temperature of the w-LEDs is 420 K. Table 2 shows the luminous intensity of SLAB:15 mol% Dy^3+^ phosphors and Dy^3+^ ion activated phosphors at 420 K. The comparison results indicate that the luminescence intensity of SLAB:15 mol% Dy^3+^ phosphor at 420 K is significantly higher than that of the reported Dy-activated phosphors. The above results indicate that the synthesized SLAB:15 mol% Dy^3+^ phosphor has excellent thermal stability and has potential advantages in w-LEDs applications [41].

The integral emission intensity of SLAB:15 mol% Dy^3+^ phosphor at 380 K is higher than 300 K. This phenomenon has also been observed in studies of BaLa_4_Si_3_O_13_:Dy^3+^, Eu^3+^, and Ca_18_Li_3_Bi(PO_4_)_14_:Eu^3+^ phosphors, and can be explained by lattice defects [42,43]. When electrons transition from the ground state to an excited state, some of the electrons in the excited state return to the ground state, while others are captured by lattice defects. As the temperature rises, some electrons trapped by lattice defects return to the ground state along with other excited electrons, thereby enhancing the emission intensity. As the temperature continues to rise, the captured excited state electrons fully return to the ground state, and thermal quenching resumes its normal state. The cooled emission spectrum of the SLAB:15 mol% Dy^3+^ phosphor is shown in Figure 8c. As the temperature decreases from 480 K to 300 K, the emission intensity gradually increases. This result has also been observed in published studies [43].

**Table 2:** Luminous intensity of different phosphors at 420 K.

Phosphors	Luminous Intensity at 420 K	References
SLAB:Dy^3+^	99.80%	This work
La_5_NbMo_2_O_16_:Dy^3+^	92.71%	[44]
La_1.96_LiNbO_6_:0.04Dy^3+^	83.00%	[30]
Na_3_La(PO_4_)_2_:Dy^3+^	52.00%	[45]
Ba_2_Gd_5_B_5_O_17_:Dy^3+^	<52.20%	[46]

To further analyze the thermal stability of SLAB:15 mol% Dy^3+^ phosphors, the activation energy (*E*_a_) of the phosphors can be determined by the Arrhenius Equation (5) [47,48].

(5) $$I_{T}=\frac{I_{0}}{1+cexp^{\left(\frac{-E_{a}}{kT}\right)}}$$

**Figure 9:**
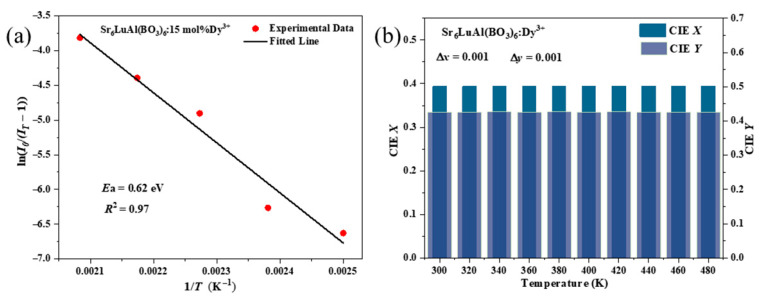
(**a**) The plot of $\ln\left[\left(I_{0}/I\right)-1\right]$ versus 1T of SLAB:*x*Dy^3+^. (**b**) Variation in CIE chromaticity coordinates of SLAB:15 mol% Dy^3+^ phosphor with temperature.

$I_{0}$ is the integral photoluminescence intensity at 300 K and $I_{T}$ is the integrated photoluminescence intensity at different test temperatures. *k* is the Boltzmann constant and *c* is the physical constant. According to Figure 9a, which shows the relationship between $\ln\left[\left(I_{0}/I\right)-1\right]$ and 1T, the activation energy (*E*_a_) of the SLAB:15 mol% Dy^3+^ phosphor can be obtained as 0.62 eV. The activation energy of the sample is superior to that of some previously reported phosphors, such as Mg_7_Ga_2_GeO_12_:Cr^3+^ (0.236 eV), YPO_4_:Eu^3+^: Sm^3+^ (0.205 eV), Ba_2_NaNb_5_O_15_:Cr^3+^ (0.265 eV), and Ca_3_WO_6_:Bi^3+^ (0.310 eV) [49,50,51,52]. This is consistent with the results shown in Figure 8, indicating that the synthesized SLAB:15 mol% Dy^3+^ phosphor exhibits excellent thermal stability.

Figure 9b shows the CIE chromaticity coordinates of SLAB:15 mol% Dy^3+^ phosphors as a function of temperature. As the temperature rises from 300 K to 480 K, the *x* and *y* values of the chromaticity coordinates remain almost unchanged, indicating that the luminous color of the sample does not change with temperature and exhibits good color stability. It illustrated that the obtained SLAB:15 mol% Dy^3+^ phosphor has excellent color stability.

**Figure 10:**
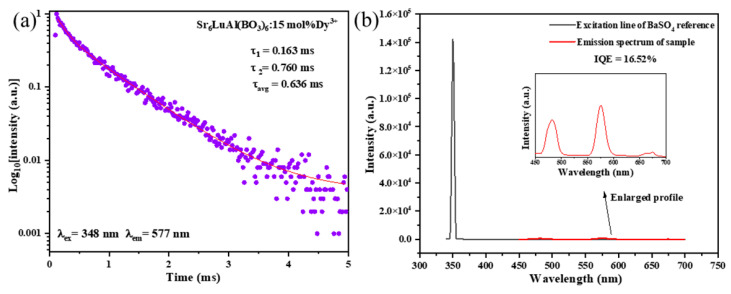
(**a**) Luminescence decay curve and (**b**) quantum yield of SLAB:15 mol% Dy^3+^ phosphor. (The excitation line of the BaSO_4_ reference and the emission spectrum of the SLAB:15 mol% Dy^3+^ phosphor collected using an integrating sphere. Inset: An enlarged profile of the emission spectrum from 450 nm to 700 nm.).

Under 348 nm excitation, the luminescence decay curve of the SLAB:15 mol% Dy^3+^ phosphor is shown in Figure 10a. According to luminescence decay theory, the luminescence lifetime of the phosphor can be obtained using Equation (6) [53].

(6) $$I_{\left(t\right)}=I_{0}+A_{1}exp(-\frac{t}{\tau_{1}})+A_{2}exp(-\frac{t}{\tau_{2}})$$

where *A*_1_ and *A*_2_ are constants, *I*_(*t*)_ is the luminescence intensity at time *t*, $\tau_{1}$ and $\tau_{2}$ are the components of fast and slow life. The average luminescence decay lifetime can be obtained from Equation (7) [54,55].

(7) $$\tau_{\mathrm{avg}}=\frac{A_{1}\tau_{1}^{2}+A_{2}\tau_{2}^{2}}{A_{1}\tau_{1}+A_{2}\tau_{2}}$$

The average lifetimes of SLAB:15 mol% Dy^3+^ phosphors were calculated to be 0.636 ms. The internal quantum yield (IQE) is an important parameter for the luminous efficiency of phosphors. According to the test results in Figure 10b, the internal quantum yield of the SLAB:15 mol% Dy^3+^ phosphor is 16.52%, indicating that this phosphor can be used to prepare w-LEDs.

**Figure 11:**
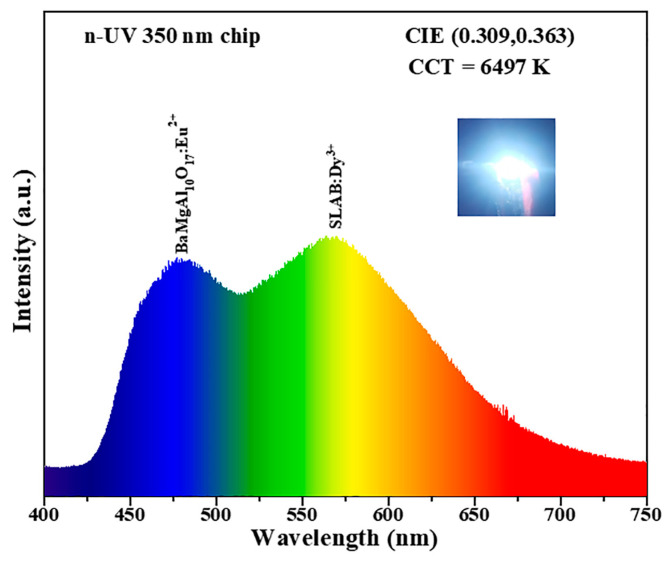
Emission spectrum of the prepared w-LEDs device. (Inset: photograph of the as-fabricated w-LEDs device when it is turned on.).

Figure 11 shows the luminescence spectrum of the prepared w-LEDs devices. The fabricated w-LEDs integrate a commercial blue phosphor BaMgAl_10_O_17_:Eu^2+^ and newly prepared yellow-emitting SLAB:Dy^3+^ phosphor. Under the excitation of a 350 nm n-UV chip, the prepared w-LEDs emit bright white light. The inset shows a real-life image of the w-LEDs emitting light. The CIE chromaticity coordinates are precisely determined as (0.309, 0.363), with a correlated color temperature (CCT) of 6497 K. Therefore, the synthesized SLAB:Dy^3+^ phosphors are expected to be applied to w-LEDs.

## 3. Materials and Methods

The yellow-emitting SLAB:*x*Dy^3+^ (*x* = 1, 3, 5, 10, 15, 20, and 30 mol%) phosphors were prepared via high-temperature solid-state reaction. Lu_2_O_3_ (AR), Al_2_O_3_ (AR), SrCO_3_ (AR), H_3_BO_3_ (AR), and Dy_2_O_3_ (AR) are the raw materials for the synthetic sample and have not undergone any pretreatment. The synthesis reaction proceeds according to the following equation:

$$\begin{array}{l} 6SrCO_{3}+0.5\left(1-x\right)Lu_{2}O_{3}+0.5xDy_{2}O_{3}+0.5Al_{2}O_{3}+\\ 6H_{3}BO_{3}\overset{1000{}^{\circ}C,4h}{\to }Sr_{6}Lu_{1-x}Dy_{x}Al{\left(BO_{3}\right)}_{6} \end{array}$$

Each ingredient was weighed according to the stoichiometric ratio in the chemical reaction equation, thoroughly ground in an agate mortar, and transferred to an alumina crucible. Next, calcination for 4 h in a muffle furnace at 1000 °C. After calcination, the samples were naturally cooled to room temperature and fully ground in an agate mortar for subsequent test characterization.

The phase purity of the synthesized powders was assessed using X-ray diffraction (XRD) analysis, employing a Rigaku Ultima IV (Rigaku Corporation, Tokyo, Japan) diffractometer with a Cu Kα X-ray source operated at 40 mA and 40 kV. The absorption spectra of the samples were measured with a UV-visible spectrophotometer UV-2600i (Shimadzu, Kyoto, Japan) equipped with an integrating sphere. Particle size distribution of the phosphor was determined by a particle size analyzer (Malvern, MS2000, Malvern City, UK). Surface morphology and elemental distribution of the samples were analyzed with SU5000 (Hitachi, Tokyo, Japan) scanning electron microscope and energy dispersive X-ray spectrometer Xplore 30 (Oxford Instruments, Oxford, UK). The fluorescence spectra of the samples were examined using a FLS-980 fluorescence spectrometer (Edinburgh Instruments, Livingston, UK). The internal quantum efficiency (IQE) of the synthesized phosphors was measured by FLS-1000 spectrometer (Edinburgh Instruments, Livingston, UK) at room temperature. The thermal stability of the sample was tested using an F-4600 (Hitachi, Tokyo, Japan) fluorescence spectrometer.

## 4. Conclusions

In this study, a series of yellow-emitting SLAB:Dy^3+^ phosphors were synthesized through traditional high-temperature solid-state reactions. The doped Dy^3+^ ions did not alter the crystal structure of the SLAB matrix. The synthesized SLAB:Dy^3+^ phosphors exhibit an irregular shape with a particle size of approximately 5.5 μm. Under excitation at 348 nm n-UV, the synthesized SLAB:Dy^3+^ phosphors exhibit the strongest emission at 577 nm. The optimal doping concentration for Dy^3+^ ions is 15 mol%. Beyond the optimal doping concentration of Dy^3+^ ions, the luminescence intensity gradually decreases due to the effect of concentration quenching. SLAB:Dy^3+^ phosphors have excellent thermal stability, with an activation energy *E*_a_ = 0.62 eV, and can maintain 100% of their initial luminous intensity at 380 K. In addition, the doping concentration of Dy^3+^ ions and temperature have little effect on the color of SLAB:Dy^3+^ phosphors, which are concentrated in the yellow region. The CCT of the w-LEDs device prepared with the SLAB:Dy^3+^ phosphor is 6497 K and the CIE chromaticity coordinate is (0.309, 0.363). The above conclusions indicate that SLAB:Dy^3+^ phosphors have potential application value in the preparation of w-LEDs.

## Figures and Tables

**Table 1:** The weight and atomic percentage of SLAB:15 mol% Dy^3+^ sample by elemental mapping test.

Elements	Weight (%)	Atomic (%)
O	25.53	52.30
B	13.40	25.45
Al	2.55	3.18
Lu	8.53	2.80
Sr	44.88	15.85
Dy	5.12	0.42
Sum	100	100

## Data Availability

The original contributions presented in the study are included in the article, further inquiries can be directed to the corresponding authors.
